# The complete chloroplast genome sequence of a popular ornamental plant *Calibrachoa hybrida* (Solanaceae: Petunioideae)

**DOI:** 10.1080/23802359.2020.1823257

**Published:** 2020-09-18

**Authors:** Guofeng Liu, Miaomiao Sun, Peishan Zou, Wei Zhang, Jianzhong Ni

**Affiliations:** Department of Botany, Guangzhou Institute of Forestry and Landscape Architecture, Guangzhou, China

**Keywords:** *Calibrachoa hybrida*, ornamental plant, Petunioideae, chloroplast genome, phylogenetic analysis

## Abstract

*Calibrachoa hybrida* is a popular ornamental plant with colorful flowers. We present here the complete chloroplast genome sequence of *C. hybrida*. With a total length of 156,099 bp, it is comprised of a large single-copy (LSC) region of 86,695 bp and a small single-copy (SSC) region of 18,694 bp separated by two inverted repeats (IRs) of 25,355 bp for each. A total of 132 genes were identified, consisting of 87 protein-coding genes, 37 tRNA genes, and eight rRNA genes. Phylogenetic analysis using complete chloroplast genomes clustered *C. hybrida* together with petunia into the subfamily Petunioideae within the family Solanaceae.

Calibrachoa (*Calibrachoa hybrida*) refers to the horticultural hybrids originated from the hybridization of wild *Calibrachoa* species (Kishimoto et al. 2019), which belongs to the Solanaceae (nightshade) family that contains many important crops such as tobacco (*Nicotiana tabacum*), tomato (*Solanum lycopersicum*), potato (*Solanum tuberosum*), and petunia (*Petunia hybrida*). Genetically, calibrachoa is closely related to petunia, the most popular bedding plant worldwide (Van der Krol and Immink 2016) and a model species for plant biology research (Vandenbussche et al. 2016). In recent decades, calibrachoa has become a popular ornamental plant all over the world, with more colorful and smaller flowers than petunia. In contrast to petunia in which both the genome and complete chloroplast (cp) genome has been sequenced (Bombarely et al. 2016; Wong et al. 2019), however, we know very little about calibrachoa’s genetic information, which hinders its genetic improvement and application. Herein, we report the complete chloroplast genome of calibrachoa, which lay the foundation for its phylogenetic studies, chloroplast transformation, and somatic hybrid identification (Power et al. 1980; Mäder and Freitas 2019).

*Calibrachoa hybrida* ‘Aloha Gold’ was obtained from the flower market of Guangzhou, China and stored in the Germplasm Resource Nursery of Ornamental Plants, Guangzhou Institute of Forestry and Landscape Architecture (GIFLA) (N113°20’25”, E23°13’47”). Genomic DNA was extracted from fresh leaves through modified CTAB method (Doyle JJ and Doyle JL 1987) and purified to construct a ∼400-bp insert size DNA library. High-throughput sequencing with paired-end 150 bp was performed on an Illumina Novaseq platform. Approximately 9.6 Gb of raw data was generated and then assembled by GetOrganelle (Jin et al. 2020). The genome was annotated using GeSeq (Tillich et al. 2017), followed by manual correction and confirmation.

The complete chloroplast genome of *C. hybrida* (GenBank accession number: MT644126) was 1,56,099 bp in length constituting a typical quadripartite circle: a large single-copy (LSC) region (86,695 bp), a small single-copy (SSC) region (18,694 bp), and a pair of inverted repeats (IRs) (25,355 bp for each). A total of 132 genes were predicted, including 87 protein-coding genes, 37 tRNA genes, and eight rRNA genes, therein 12 protein-coding genes, 14 tRNA genes, and all rRNA genes locate in the IRs. The overall GC content for the cp genome is 37.83%.

To clarify the phylogenetic position of *C. hybrida*, a maximum likelihood (ML) tree was constructed with the chloroplast genomic sequences of 21 species from three orders (Gentianales, Lamiales, and Solanales) based on GTRGAMMA substitution model (Stamatakis 2014) with 1000 bootstrap replicates after aligned with MAFFT v7.307 (Katoh and Standley 2013). As shown in Figure 1, *C. hybrida* is posited sister to *Petunia hybrida* within the Solanaceae family. In the phylogenetic tree, *C. hybrida* and *P. hybrida* form a monophyletic lineage (Petunioideae) that split away from the branch giving rise to the Nicotianodeae and Solanoideae. This is consistent with the result of phylogenetic analysis based on genomic data (Bombarely et al. 2016).

**Figure 1.**
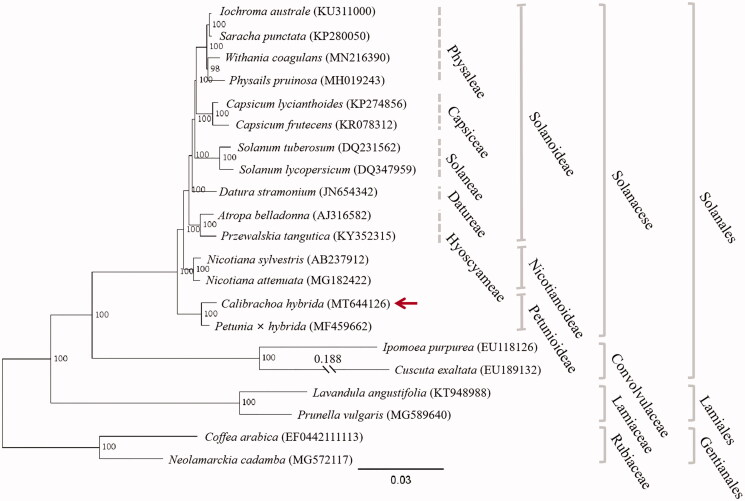
Maximum likelihood tree showing the phylogenetic position of *Calibrachoa hybrida* (arrowed) based on the complete chloroplast genome sequences of 21 species from Solanales, Lamiales, and Gentianales, with *Coffea arabica* and *Neolamarckia cadamba* as outgroup. Bootstrap values (1000 replicates) are indicated at nodes. Distance shown for truncated branches. Sequences retrieved from GenBank. Scale bar: substitutions per site.

## Data Availability

The data that support the findings of this study are openly available in NCBI at https://www.ncbi.nlm.nih.gov/nuccore/MT644126.
